# Hematological Toxicities of Concurrent Chemoradiotherapies in Head and Neck Cancers: Comparison Among Cisplatin, Nedaplatin, Lobaplatin, and Nimotuzumab

**DOI:** 10.3389/fonc.2021.762366

**Published:** 2021-10-21

**Authors:** Qiuji Wu, Chunmei Zhu, Shuyuan Zhang, Yunfeng Zhou, Yahua Zhong

**Affiliations:** Department of Radiation and Medical Oncology, Hubei Key Laboratory of Tumor Biological Behaviors, Hubei Cancer Clinical Study Center, Zhongnan Hospital of Wuhan University, Wuhan, China

**Keywords:** concurrent chemoradiotherapy, platinum, nimotuzumab, hematological toxicity, head and neck cancer

## Abstract

**Background:**

Cisplatin-based concurrent chemoradiotherapy is standard of care for locally advanced head and neck cancers (LAHNC). Nedaplatin, lobaplatin and nimotuzumab have shown anti-cancer effect with less gastrointestinal toxicity and nephrotoxicity. However, the profile of hematological toxicities of these agents in combination with radiotherapy has not been fully illustrated.

**Methods:**

We retrospectively collected the clinical data of consecutive LAHNC patients treated by cisplatin-, nedaplatin-, lobaplatin-, and nimotuzumab-based concurrent chemoradiotherapy. Routine blood cell counts were obtained every 4 to 7 days. Hematological toxicities were graded according to the Common Terminology Criteria for Adverse Events (CTCAE) Version 5.0.

**Results:**

A total of 181 eligible LAHNC patients were assigned to nimotuzumab group (n = 34), cisplatin group (n = 52), nedaplatin group (n = 62) or lobaplatin group (n = 33). Among the four groups, nimotuzumab group displayed lightest hematological toxicities, followed by cisplatin group, nedaplatin group, and lobaplatin group. Lobaplatin was more likely to produce grade 3/4 leukopenia compared with cisplatin (48.5% *vs* 25.0%). Compared with cisplatin, nedaplatin and lobaplatin were more likely to cause grade 3/4 thrombocytopenia (nedaplatin 19.4% *vs* cisplatin 3.8%; lobaplatin 30.3% *vs* cisplatin 3.8%). Similarly, nimotuzumab group showed highest nadir levels among the four groups, followed by cisplatin, nedaplatin, and lobaplatin group. Moreover, concurrent platinum treatment and induction chemotherapy were risk factors of developing grade 3/4 hematological toxicities.

**Conclusion:**

Nimotuzumab-based concurrent chemoradiotherapy in head and neck cancers produced the lightest hematological toxicities, followed by cisplatin, nedaplatin, and lobaplatin. Patients should be given specific attention during concurrent chemoradiotherapy, particularly in the presence of previous induction chemotherapy.

## Introduction

Head and neck cancers account for the seventh most common malignancy worldwide with 890,000 estimated new cases and 450,000 estimated deaths in 2018 (1). Although early head and neck cancers are curable with radical surgery or radiotherapy and have favorable prognosis, more than 60% of patients present with advanced disease that require multidisciplinary treatment and carry high risk of local regional recurrence and distant metastasis (2). For inoperable locally advanced head and neck cancer (LAHNC) patients, high-dose cisplatin-based concurrent chemoradiotherapy (CCRT) remains standard of care (3–5). However, this treatment modality is associated with increased toxicities and is less tolerated in elderly patients or patients with poorer performance status. Main toxicities include Mucositis, hematological, gastrointestinal, renal and neurological toxicities, etc. Therefore, other less toxic platinum-based chemoradiotherapy and alternative concurrent regimens have also been actively investigated.

Nedaplatin is a cisplatin analog with decreased nephrotoxicity and gastrointestinal toxicity (6). Nedaplatin-based concurrent chemotherapy showed comparable effectiveness to cisplatin in terms of disease control and patient survival in nasopharyngeal carcinoma and head and neck cancers (7–9). Importantly, while nedaplatin led to less gastrointestinal toxicities, nephrotoxicity, ototoxicity, and neurotoxicity compared with cisplatin, more hematologic toxicities such as grade 3/4 neutropenia and thrombocytopenia were seen in nedaplatin-treated patients (7, 8). Lobaplatin is another platinum compound showing anti-tumor activity in multiple solid tumors such as breast cancer, small-cell lung cancer, and hepatocellular carcinoma (10–12). Lobaplatin-based induction chemotherapy followed by lobaplatin-radiotherapy showed comparable survival outcomes but less acute toxicities than cisplatin-based concurrent chemoradiotherapy in locally advanced nasopharyngeal carcinoma and might be a promising alternative to cisplatin-based treatment (13). Importantly, leucopenia, neutropenia, and gastrointestinal toxicities were more documented in cisplatin-treated patients (14). For elderly patients or those who are intolerant to platinum-based concurrent chemotherapy, anti-EGFR antibodies have also shown semblable effectiveness and could be used as concurrent treatments in head and neck cancers (15–21). Cetuximab is a chimeric anti-EGFR monoclonal antibody. Although cetuximab-based CCRT showed superiority than radiotherapy alone in LAHNC (22), it did not outperformed cisplatin-based CCRT (23). Nimotuzumab is a humanized anti-EGFR antibody. The addition of nimotuzumab to weekly cisplatin-based concurrent chemoradiotherapy resulted in improved progression-free survival, locoregional control in locally advanced head and neck cancer (19, 20, 24). In head and neck cancer patients unfit for chemoradiotherapy, nimotuzumab plus radiotherapy yielded higher complete response and favorable overall survival compared with radiotherapy alone (25).

Although multiple alternative approaches are under investigation, cisplatin-based CCRT still remain the mainstay for LAHNC. Whereas in cases unfit for cisplatin treatment, platinum-analogs and anti-EGFR antibodies might be an attractive choice. However, direct comparison of toxicity profiles among these drugs has not been performed. In this study, we aimed to compare the hematological toxicities in nimotuzumab-, cisplatin-, nedaplatin- and lobaplatin-based concurrent chemoradiotherapy in locally advanced head and neck cancer.

## Materials and Methods

### Patient Cohort

We conducted this retrospective clinical observational study at Zhongnan Hospital of Wuhan University. Patients with locally advanced head and neck cancer sequentially treated with concurrent chemoradiotherapy with or without prior induction chemotherapy from January 2017 to October 2020 were enrolled in this study. All patients had a complete history and physical examination, endoscopic and imaging evaluation, complete blood test, biochemical profile. The inclusion criteria were as follows: (1) patients with histologically confirmed, nasopharyngeal cancer, oropharyngeal cancer, nasal sinus cancer, oral cancer and other rarer head and neck cancers; (2) adequate hematological function (white blood cell count ≥ 4*10^9^/L, platelet count ≥ 100*10^9^/L, and hemoglobin ≥ 90 g/L); (3) adequate renal function (creatinine clearance ≥60 mL/min); (4) adequate hepatic function (serum bilirubin, alanine amino transferase, and aspartate aminotransferase ≤ 2.0 times the upper limit of normal); and (5) Karnofsky score ≥ 70. The exclusion criteria included: (1) previous radiotherapy for head and neck cancers; (2) previous malignancies; (3) uncontrolled medical or psychiatric disease; and (4) pregnancy or lactation. The study was conducted in accordance with the Declaration of Helsinki and the International Conference on Harmonization Good Clinical Practice guidelines, and was approved by the ethics committee of the Zhongnan Hospital of Wuhan University (No. 2019130). Informed consent was obtained from all included patients.

### Radiotherapy

All patients received conventional fractionated (2Gy per fraction, 5 fractions per week, from Monday to Friday) simultaneous integrated boost intensity-modulated radiotherapy (IMRT) to the primary lesions and neck lymph node areas. Radiation doses were given 68-70Gy to gross tumor volumes, 60-66Gy to high-risk cervical lymphatic draining areas, 54Gy to low-risk lymphatic draining areas.

### Chemotherapy

#### Induction Chemotherapy

Induction chemotherapy prior to concurrent chemoradiotherapy mainly consisted of DC regimen (docetaxel 75 mg/m^2^ on day 1 plus cisplatin or nedaplatin 75 mg/m^2^ on day 1; every three weeks). Other regimens included DCF regimen (docetaxel 75 mg/m^2^ day on day 1 plus cisplatin or nedaplatin 75 mg/m^2^ on day 1, and 5-FU 750 mg/m^2^ from day 1 to day 5; every three weeks) and GP regimen (gemcitabine 1 g/m^2^ on day 1 and day 8, plus cisplatin or nedaplatin 75 mg/m^2^ on day 1; every three weeks) according to patients’ disease, age, performance status and comorbidities.

#### Concurrent Chemotherapy

Concurrent chemotherapy regimens were chosen according to patients’ age, performance status and comorbidities. In patients who were tolerable to cisplatin treatment, cisplatin was given either tri-weekly 80-100 mg/m^2^ or weekly fixed dose of 50 mg. For elderly patients or those with reduced tolerance to cisplatin, the following regimens were used instead: (1) tri-weekly nedaplatin 75-100 mg/m^2^, weekly nedaplatin 50 mg; (2) weekly nimotuzumab 100 mg or 200 mg; (3) or tri-weekly lobaplatin 35 mg/m^2^ or 50 mg/m^2^, according to physicians’ choice during the course of radiotherapy.

### Hematopoietic Support Treatment

Prophylactic use of granulocyte colony-stimulating factor, erythropoietin or thrombopoietin was not allowed. When hematological toxicity occurred, patients were given hematopoietic support treatment. Recombinant human G-CSF or GM-CSF was given when the white blood cell (WBC) count was < 3.0*10^9^/L. Recombinant human erythropoietin was given when the hemoglobin (HGB) level was < 90 g/L. Red blood cell transfusion was used when the hemoglobin level was < 60 g/L. Recombinant human thrombopoietin or IL-11 was given when the platelet (PLT) count was < 75*10^9^/L. Platelet transfusion was used when the platelet count was < 10*10^9^/L.

### Acute Hematological Toxicity Evaluation

Blood routine tests were performed every 4-6 days during the course of concurrent chemoradiotherapy. Hematological toxicities evaluation was based on the “Common Terminology Criteria for Adverse Events” (CTCAE, version 5.0) (26). The lowest values of studied items, events of febrile neutropenia and requirement of hematopoietic support treatment and blood cell transfusion were also noted and analyzed.

### Statistical Analysis

IBM SPSS Statistics (Version 22.0. Armonk, NY: IBM Corp), GraphPad (Version 6.0, GraphPad Software Inc, San Diego, CA, USA) were used for data analysis and visualization. Categorical variables were presented as frequencies and percentages and quantitative data were described as either mean (standard deviation, SD) or median (interquartile range, IQR). χ² test or Fisher’s exact test for categorical variables and Kruskal-Wallis tests (with Bonferroni correction for pairwise comparisons among multiple groups) for continuous variables were applied. Binary logistic regression was performed to assess the association of age, gender, BMI, concurrent drug, and induction regimen with grade 3-4 hematological toxicities. All statistical tests were two-sided, and a P value < 0.05 was considered statistically significant.

## Results

### Patient Characteristics

From January 2017 to October 2020, a total of 181 eligible patients were included in this study. Most patients were male (n=131, 72.4%). The median age was 52 (12–77) years old. Cancer types were consisted of nasopharyngeal carcinoma (n=88, 48.6%), oral cavity cancers (n=49, 27.1%), nasal cavity and sinus tumors (n=17, 9.4%), oropharyngeal cancer (n=12, 6.6%) and other rarer HNC (n=15, 8.3%). Most patients were with stage III-IVB diseases (n=162, 89.5%). Near half of the patients (n=107, 59.1%) received induction chemotherapy, mostly using DC regimen (n=66, 61.7%). The baseline characteristics of the four groups were listed in **Table 1**.

**Table 1:** Characteristics of LAHNC patients.

Characteristic	All Patients n = 181	Nimotuzumab n = 34	Cisplatin n = 52	Nedaplatin n = 62	Lobaplatin n = 33	*p* value
**Age,y**						0.005
Median (range)	52 (12-77)	60 (32-77)	52 (18-69)	49 (12-66)	53 (34-67)	
**Gender**						0.225
Female	50 (27.6%)	10 (29.4%)	9 (17.3%)	19 (30.6%)	12 (36.4%)	
Male	131 (72.4%)	24 (70.6%)	43 (82.7%)	43 (69.4%)	21 (63.6%)	
**Type of tumor**						0.254
Nasopharyngeal carcinoma	88 (48.6%)	16 (47.1%)	21 (40.4%)	37 (59.7%)	14 (42.4%)	
Oropharyngeal cancer	12 (6.6%)	2 (5.9%)	2 (3.8%)	6 (9.7%)	2 (6.1%)	
Nasal cavity and sinus tumors	17 (9.4%)	2 (5.9%)	10 (19.2%)	2 (3.2%)	3 (9.1%)	
Oral cavity cancers	49 (27.1%)	10 (29.4%)	15 (28.8%)	14 (22.6%)	10 (30.3%)	
Other HNSCC^*^	15 (8.3%)	4 (11.8%)	4 (7.7%)	3 (4.8%)	4 (12.1%)	
**Induction chemotherapy regimens**						0.170
DC*	66 (36.5%)	8 (23.5%)	16 (30.8%)	29 (46.8%)	14 (39.4%)	
Others	41 (22.7%)	10 (29.4%)	13 (25.0%)	14 (22.6%)	4 (12.1%)	
**Cycles of induction chemotherapy**						0.094
0	74 (40.9%)	18 (52.9%)	23 (44.2%)	19 (30.6%)	16 (48.5%)	
1-2 cycles	59 (32.6%)	7 (29.4%)	14 (26.9%)	25 (40.3%)	13 (39.4%)	
3-4 cycles	45 (23.8%)	10 (23.5%)	14 (26.9%)	18 (29.0%)	3 (9.1%)	
>4 cycles	3 (1.7%)	1 (2.9%)	1 (1.9%)	0 (0%)	1 (3.0%)	

^*^Other HNSCC: hypopharyngeal cancer, laryngeal cancer and cancers of the salivary glands.

*DC, docetaxel plus cisplatin or nedaplatin.

During concurrent chemoradiotherapy, patients were assigned to nimotuzumab group (n=34, 18.8%), cisplatin group (n=52, 28.7%), nedaplatin group (n=62, 34.3%) or lobaplatin group (n=33, 18.2%). Besides, in the lobaplatin group, 4 patients were initially treated with cisplatin and then shifted to lobaplatin, 6 patients were initially treated with nedaplatin and then shifted lobaplatin, and 23 patients with lobaplatin alone. All patients completed at least one cycle of concurrent chemotherapy.

In terms of treatment completion, for weekly treated patients, the median completed cycles were 6 (range: 3-8), 4 (range: 1-6) and 4 (range: 1-5) for nimotuzumab, nedaplatin and cisplatin, respectively. No patient completed 6 cycles of cisplatin. On the other hand, 14.0% of patients treated with nedaplatin and 52.9% of patients treated with nimotuzumab completed ≥ 6 cycles of treatment. For tri-weekly treated patients, the majority of patients could complete 2 cycles of treatment. No patient completed 3 cycles of cisplatin. Similarly, only 5.3% and 9,1% patient completed 3 cycles of nedaplatin and lobaplatin, respectively (**Supplementary Table S1**).

### Concurrent Chemoradiotherapy-Induced Hematological Toxicities

**Figure 1:**
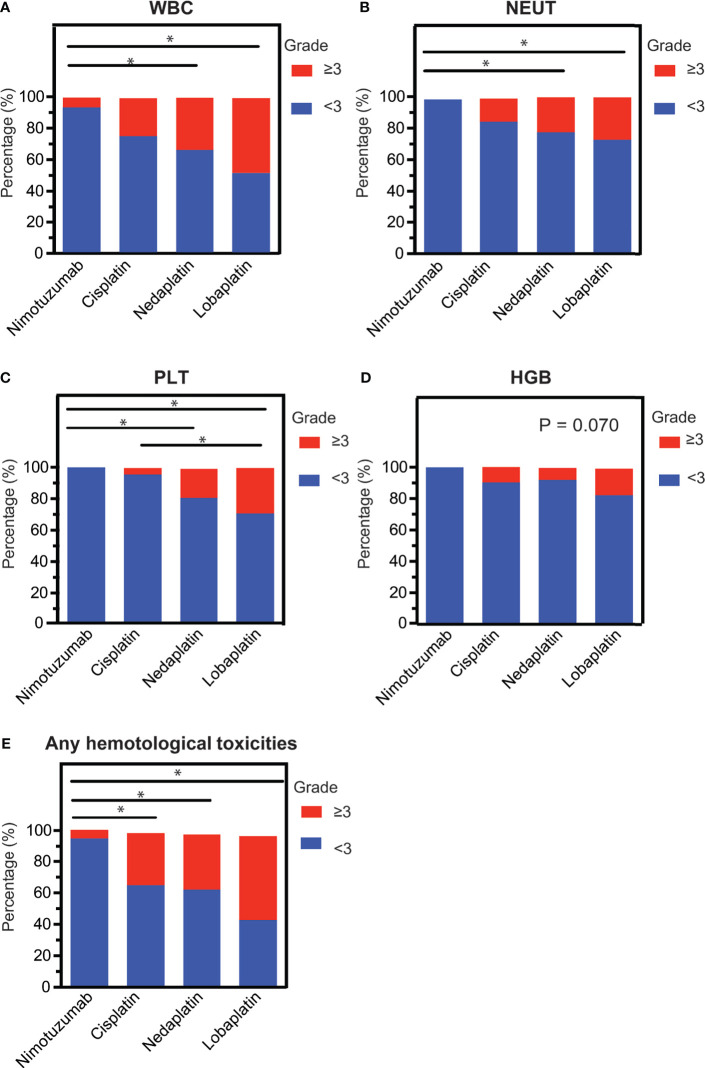
The intra-grade ratios of hematological toxicities. **(A)** Leukopenia, **(B)** neutropenia, **(C)** thrombocytopenia, **(D)** erythropenia, and **(E)** any hematological toxicity among LAHNC patients treated with nimotuzumab-, cisplatin-, nedaplatin- and lobaplatin-based concurrent chemoradiotherapy. WBC, white blood cell; NEUT, neutrophil; PTL, platelet; HGB, hemoglobin. *P < 0.05.

To determine the hematological toxicity profiles of the four concurrent regimens, we examined their impacts on patients’ peripheral white blood cell, neutrophil, hemoglobin, and platelet during the course of CCRT. Overall, lobaplatin was more likely to induce grade 3/4 hematological toxicities, followed by nedaplatin, cisplatin, and nimotuzumab. The nimotuzumab group had the lowest frequency of grade 3/4 leukopenia, thrombocytopenia and neutropenia compared with the nedaplatin group and lobaplatin groups (P < 0.05). The cisplatin group had lower grade 3/4 grade thrombocytopenia compared with the lobaplatin group (P < 0.05). However, there was no significant difference between the nedaplatin group and the lobaplatin group (**Figure 1**, **Table 2**).

**Table 2:** Concurrent chemoradiotherapy-induced myelosuppression in LAHNC patients.

Toxicities*	All Patients n=181	Nimotuzumab n=34	Cisplatin n=52	Nedaplatin n=62	Lobaplatin n=33	*p* value
**White blood cell**						
Any Grade	166 (91.7%)	28 (82.4%)	47 (90.4%)	39 (95.2%)	32 (97%)	0.002
Grade 3-4	52 (28.7%)	2 (5.9%)	13 (25%)	21 (33.9%)	16 (48.5%)	0.001
**Neutrophil**						
Any Grade	119 (65.7%)	11 (32.4%)	36 (69.2%)	44 (71%)	28 (84.8%)	0.001
Grade 3-4	31 (17.2%)	0 (0%)	8 (15.4%)	14 (22.6%)	9 (27.3%)	0.003
**Hemoglobin**						
Any Grade	99 (54.7%)	15 (44.1%)	32 (61.5%)	30 (48.4%)	22 (66.7%)	0.318
Grade 3-4	16 (8.9%)	0 (0%)	5 (9.6%)	5 (8.1%)	6 (18.2%)	0.070
**Platelet**						
Any Grade	71 (39.2%)	4 (21.8%)	16 (30.8%)	29 (46.8%)	22 (66.7%)	<0.001
Grade 3-4	24 (13.3%)	0 (0%)	2 (3.8%)	12 (19.4%)	10 (30.3%)	0.001
**Hematologic**						
Any Grade	171 (94.5%)	30 (88.2%)	49 (94.2%)	60 (96.8%)	32 (97.0%)	0.389
Grade 3-4	63 (34.8%)	2 (5.9%)	18 (34.6%)	24 (38.7%)	19 (57.6%)	<0.001

*According to CTCAE criteria (CTCAE, version 5.0).

### Impact on the Nadir of Blood Cell and Hemoglobin Counts

**Figure 2:**
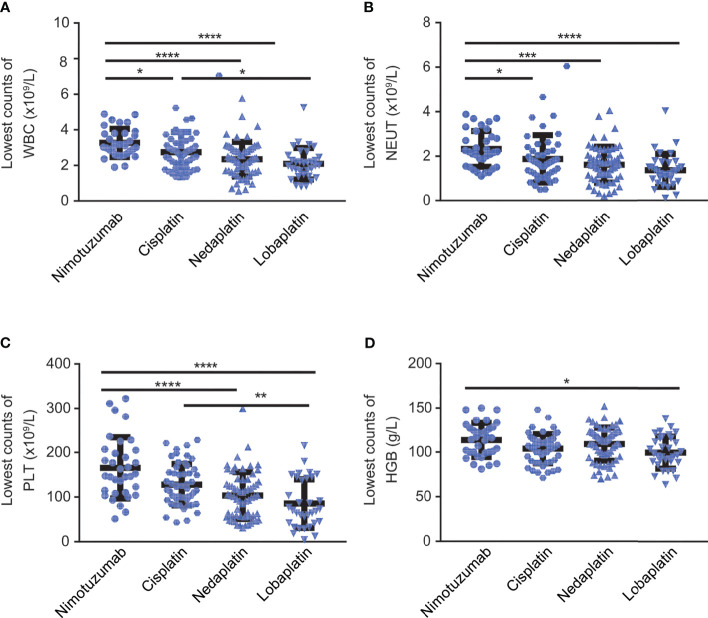
The lowest counts of blood cell and hemoglobin counts during CCRT. Lowest count of **(A)** white blood cells, **(B)** neutrophils, **(C)** platelets, and **(D)** hemoglobin among LAHNC patients treated with nimotuzumab-, cisplatin-, nedaplatin- and lobaplatin-based concurrent chemoradiotherapy. WBC, white blood cell; NEUT, neutrophil; PTL, platelet; HGB, hemoglobin. *P < 0.05, **P < 0.01, ***P < 0.001, ****P < 0.0001.

In terms of the nadir of white blood cell, neutrophil, platelet and hemoglobin counts detected during concurrent chemoradiotherapy, the nimotuzumab group had the highest levels of all these parameters among the four groups. On the contrary, the lobaplatin group had the lowest values in all aspects. Specifically, the nimotuzumab group had significantly higher values of the nadir of white blood cell count, neutrophils cell count, and platelets count compared with the nedaplatin group and lobaplatin group (P < 0.001). There was also a higher value of the nadir of red blood cell count in the nimotuzumab group than in the lobaplatin group (P < 0.05). In addition, the nadirs of white blood cell count and platelet count were higher in the cisplatin group than in the lobaplatin group (P < 0.05). However, in terms of the lowest blood cell count, there was no significant difference between the cisplatin group and the nedaplatin group, between the nedaplatin group and lobaplatin group (**Figure 2**, **Table 3**).

**Table 3:** Nadir of blood cell counts during concurrent chemoradiotherapy in LAHNC patients.

Nadir of cell counts	Nimotuzumab n=34	Cisplatin n=52	Nedaplatin n=62	Lobaplatin n=33	*p* value
**White blood cell, 10^9^/L**					<0.001
Median (IQR)	3.1 (2.65-3.80)	2.64 (1.96-3.12)	2.37 (1.68-2.91)	2.02 (1.45-2.51)	
**Neutrophil, 10^9^/L**					<0.001
Median (IQR)	2.2 (1.54-2.94)	1.71 (1.20-2.22)	1.6 (1.08-2.06)	1.29 (0.92-1.72)	
**Hemoglobin, g/L**					<0.05
Median (IQR)	115 (99.8-127.3)	106.5 (90.3-113.8)	112.5 (96.5-123.0)	97 (89.5-116.0)	
**Platelet, 10^9^/L**					<0.001
Median (IQR)	149.5 (109.5-205.5)	121 (95.3-160.3)	102 (57.0-135.3)	70 (41.5-143.5)	

### Effect of Weekly and Tri-Weekly Concurrent Chemotherapy on Hematological Toxicities

In nimotuzumab group patient was intravenous injected once a week, five patients were given 200 mg once a week, and 29 patients received 100 mg once a week. Patients were given cisplatin at 250.0 mg (IQR 152.5-260.0 mg) in cisplatin group and nedaplatin at 250.0 mg (IQR 200.0-280.0 mg) in nedaplatin group. In lobaplatin group, patients were received lobaplatin at 100 mg (87.5-155.0 mg).

Next, we also explored whether there was different impact of weekly and tri-weekly concurrent chemotherapy on hematological toxicities. In total, 31 patients in the cisplatin group, and 19 patients in the nedaplatin group had received tri-weekly concurrent platinum treatment.

There was no significant difference in hematological toxicity between the three-week regimen and the one-week regimen of cisplatin group, however, grade 3/4 thrombocytopenia occurred more frequently in weekly treated patients in nedaplatin group compared with the tri-weekly patients (**Supplementary Table S2**).

### Febrile Neutropenia and Hematological Supportive Treatment

During concurrent chemoradiotherapy, 1 (1.9%) patient in the cisplatin group, 3 (4.8%) patients in the nedaplatin group and 3 (9.9%) patients in the lobaplatin group developed a febrile neutropenia, and were given supportive treatments including granulocyte stimulating factors, prophylactic antibiotics treatment and fluid infusions etc. The use of granulocyte stimulating factors, erythropoietin stimulating factor, platelet stimulating factor, and blood transfusion were more frequent in the nedaplatin group and the lobaplatin group. Eighteen (54.5%) patients in the lobaplatin group, 25 (40.3%) patients in the nedaplatin group, 11 (21.2%) patients in the cisplatin group and 4 patients (11.8%) in the nimotuzumab group received thrombopoietic treatment. Eight (24.2%) patients in the lobaplatin group, 7 (11.3%) patients in the nedaplatin group and 4 (7.7%) patients in the cisplatin group, 2 (5.9%) patients in the nimotuzumab group received erythropoietin treatment (**Table 4**).

**Table 4:** Febrile neutropenia, platelet infusion and requirement of growth factors during concurrent chemoradiotherapy in LAHNC patients.

Events	All Patients n=181	Nimotuzumab n=34	Cisplatin n=52	Nedaplatin n=62	Lobaplatin n=33
**Febrile neutropenia**	7 (3.9%)	0 (0%)	1 (1.9%)	3 (4.8%)	3 (9.9%)
**Platelet infusion**	3 (1.7%)	0 (0%)	0 (0%)	1 (1.6%)	2 (6.1%)
**rhTPO or IL-11 use**	58 (32.0%)	4 (11.8%)	11 (21.2%)	25 (40.3%)	18 (54.5%)
**rhEPO use**	21 (11.6%)	2 (5.9%)	4 (7.7%)	7 (11.3%)	8 (24.2%)

### Risk Factors Associated With Hematological Toxicities

To identify risk factors that would potentially contribute to the occurrence of hematological toxicities, we performed univariate binary logistic regression analysis that included age, gender, body mass index (BMI) of patients, concurrent drug, and induction regimen. Our results indicated that lobaplatin were more likely to cause grade 3/4 hematological toxicities [OR = 21.71 (95% CI: 4.44–106.12), P < 0.001], followed by nedaplatin (OR = 10.11 (95% CI: 2.22–46.08), P < 0.001], cisplatin [OR = 8.47 (95% CI: 1.82–39.46); P = 0.01] with reference to nimotuzumab. This was consistent with previous findings of our study. Induction chemotherapy (DC regimen) also rendered patients more likely to develop grade 3/4 myelosuppression (**Table 5**).

**Table 5:** Risk factor associated with grade 3-4 hematological toxicities based on binary logistic regression.

Variable	N	OR (95%CI)	*p* value
**Age**			
<=60 (ref.)	142		
>60	39	0.58 (0.26-1.28)	0.18
**Gender**			
Female (ref.)	50		
Male	131	0.65 (0.33-1.27)	0.21
**BMI**			
<=24 (ref.)	123		
>24	58	0.87 (0.45-1.69)	0.69
**CCRT**			
Nimotuzumab (ref.)	34		
Cisplatin	52	8.47 (1.82-39.46)	0.01
Nedaplatin	62	10.11 (2.22-46.08)	<0.001
Lobaplatin	33	21.71 (4.44-106.12)	<0.001
**IC regimen**			
None (ref.)	77		
DC	66	2.24 (1.11-4.5)	0.02
Others	41	0.93 (0.39-2.18)	0.86

## Discussion

Platinum-based concurrent chemotherapy showed better response rates and overall survival rates than radiotherapy alone, surgery alone or surgery plus radiotherapy in locally advanced head and neck cancers (LAHNC). However, the optimal concurrent chemotherapy regimen with or without induction chemotherapy is still inconclusive (27–30). Cisplatin-based concurrent chemoradiotherapy improved patient survival compared with radiotherapy alone in inoperable LAHNC, although it also increases toxicities such as gastrointestinal, hematological, renal and auditory toxicities (31–33). In those unfit or intolerant to cisplatin treatment, less toxic platinum analog and anti-EGFR antibodies might provide comparable effectiveness and could be alternative choices. However, the toxicity profiles and in particular the hematological toxicity differences among those treatments have not been fully demonstrated.

In this retrospective study, we found substantial differences in hematological toxicities among cisplatin-, nedaplatin-, lobaplatin-, and nimotuzumab-based concurrent chemoradiotherapy in LAHNC. Our results demonstrate that nimotuzumab had the lightest hematological toxicity, followed by cisplatin, nedaplatin, while lobaplatin had the most severe hematological toxicity. To our knowledge, this is the first study that compares these four commonly used chemotherapeutic agents in this setting.

Platinum salt was firstly developed by chemotherapist Peyrone in 1844 and since played central roles in anti-cancer chemotherapy. In 1965, Rosenberg et al. reported that cisplatin had potential anti-tumor activity. Subsequently, second-generation (carboplatin, nedaplatin) and third-generation platinums (oxaliplatin, lobaplatin) were also developed, with varied antitumor activities in different malignancies (34). In addition, these reagents were characterized with different toxicity profiles. Cisplatin (cis-dichlorodiamine platinum) was mainly metabolized by the kidneys and a large amount of oxygen free radicals were produced during the metabolic process, which could cause kidney injury. The glycolic acid on the structure of nedaplatin (Cis-glycolic acid diamine platinum) replaced two chloride ions, which had high solubility in water and thus changed the distribution in the kidneys. As a result, nedaplatin had less nephrotoxicity. In addition, nedaplatin induced lower ototoxicity and gastrointestinal toxicity (14). Lobaplatin (Chemical name: 1,2-Diamino-cyclobutane-lactate platinum) was a 1:1 diastereomers mixture of platinum complexes containing a 1,2-bis (aminomethyl) cyclobut ane stables ligand and lactic acid as the leaving group. As the latest third-generation platinum-derived anti-cancer drug, lobaplatin showed favorable antitumor activity, good solubility and stability in water. Moreover, lobaplatin carried tolerable toxicity and could potentially overcome tumor resistance to cisplatin (35). The component and structural difference among these platinum analogs gave rise to their distinct pharmaco-mechanisms and pharmacokinetic, which helped to explain their difference on hematological toxicity profiles observed in both clinical trials and real-world studies.

One of the main toxicities of platinum is dose limiting hematological toxicity. In line with our results, similar reports that compare either two of these drugs have showed varied hematological toxicities, although no direct comparison among four agents has yet been reported (7, 8, 10). Nedaplatin was more prone to induce grade 3/4 thrombocytopenia than cisplatin. In addition, grade 3 or worse adverse events of leucopenia, neutropenia and thrombocytopenia were more frequently observed in nedaplatin-treated patients than in cisplatin-treated ones (36). Likewise, lobaplatin was reported to result in more severe thrombocytopenia than cisplatin (13). In fact, during the course of lobaplatin-radiotherapy of locally advanced nasopharyngeal carcinoma, main grade 3/4 acute adverse events included thrombocytopenia, leucopenia, neutropenia, anemia (10, 37). A phase II randomized clinical trial comparing docetaxel plus lobaplatin induction chemotherapy combined with lobaplatin chemoradiotherapy *versus* TPF induction chemotherapy combined with cisplatin chemoradiotherapy in locally advanced head and neck squamous cell carcinoma (NCT03117257) is going on and might provide more convincing comparative data on their toxicity profiles.

In this study, we compared hematological toxicities of nimotuzumab-, cisplatin-, nedaplatin-, and lobaplatin-based concurrent chemoradiotherapy of head and neck cancers. Our research results show that lobaplatin had the most severe hematological toxicity, followed by nedaplatin, cisplatin and nimotuzumab. Specifically, lobaplatin was likely to produce more serious leukopenia and thrombocytopenia compared with cisplatin. The hematological toxicity of nedaplatin was more severe than that of cisplatin but lighter than that of lobaplatin. Given that there still lacks prospective comparative study showing a superiority of other platinums over cisplatin in concurrent chemoradiotherapy in LAHNC, cisplatin should be considered in priority in cases that patients have good liver and kidney function, and tolerable gastrointestinal reaction to cisplatin (5, 38). What’s more, cisplatin is economically available. However, cisplatin treatment must be supplemented with adequate prior hydration, which may lengthen the hospital stay. Cisplatin can also cause severe gastrointestinal reactions such as nausea and vomiting, leading to decreased patient compliance. In cases of intolerance to the toxicities of cisplatin, we should consider nedaplatin that induced less gastrointestinal reaction and lower liver and kidney toxicities. Although nedaplatin caused less nausea, vomiting, liver and kidney toxicities, some patients still suffered from these events. Furthermore, a small part of patients showed allergy to nedaplatin, limiting its use in many cases (39). For patients who cannot tolerate the above-mentioned two platinum salts, lobaplatin might be an alternative choice. Lobaplatin has good water solubility, broad anti-cancer spectrum, high anti-cancer activity, and no cross-resistance with other platinums. However, lobaplatin is more toxic to the hematopoietic system. Our study demonstrated highest risk of grade 3/4 hematological toxicities associated with lobaplatin. Therefore, during the use of lobaplatin in concurrent chemoradiotherapy, patients should be given closer and more frequent monitoring of white blood cells and platelets, especially for patients older than 65, and those who have undergone multiple chemotherapies and those who have experienced previous grade 3/4 myelosuppression. In addition, lobaplatin was the most expensive among the three platinum salts. For elderly patients with poor general health conditions, or those who cannot tolerate platinum-based chemotherapy, nimotuzumab with best hematological toxicity profiles could be considered. But it was quite expensive, and was not yet covered by current medical insurance system. Therefore, in patients tolerable to platinum, we still suggest platinum-based concurrent chemotherapy.

Cisplatin and nedaplatin have been commonly used clinically and the standard dose of concurrent chemotherapy is 30 mg/m^2^ once a week or 100 mg/m^2^ once every three weeks which converting to a single dose is respectively 50 mg once a week or 160 mg once every three weeks, respectively. The effectiveness and safety results of them are also relatively mature. Previous clinical trials have evaluated the toxicities of tri-weekly treatment regimen and weekly treatment regimen and found that tri-weekly treatment regimen was associated with more hematological toxicities in terms of leukopenia, neutropenia (40, 41). In our study, the cisplatin group had the more patients using tri-weekly treatment regimen, compared with nedaplatin. Grade 3/4 thrombocytopenia occurred more frequently in weekly nedaplatin-treated patients compared with tri-weekly nedaplatin-treated patients. There was no significant difference in hematological toxicity between the weekly regimen and tri-weekly regimen in cisplatin-treated patients. As for lobaplatin, the maximum tolerated dose is 60 mg/m^2^ and the recommended dose is 30-50 mg/m^2^, and the standard dose of lobaplatin in western countries is 50 mg/m^2^. Due to ethnic and geographic differences, this dose level may not be suitable for Chinese. In our center, 23 patients received a dose of 35 mg/m^2^ and 10 patients were given a dose of 50 mg/m^2^ every three weeks.

Our study further identified induction chemotherapy prior to concurrent chemoradiotherapy was a risk factor of developing grade 3/4 hematological toxicities. These results indicated that for patients who have received induction chemotherapy, closer attention should be paid concerning potential severe myelosuppression, especially when use nedaplatin or lobaplatin as concurrent drug. Given less hematological toxicity and gastrointestinal side effects, nimotuzumab could be a safe alternate. Otherwise, cisplatin could also be considered with proper management of its adverse effects.

There were some limitations in this study. First, the sample size of eligible patients in this study was small. Second, the retrospective nature made it evitable to create selection and information bias that render the study less rigorous. Therefore, these results are supposed to provide reference to clinical decision-making and should be validated in prospective randomized trials. In addition, we did not take into account of other frequent toxicities but particularly focused on hematological side effect. More comprehensive analysis on treatment effectiveness, toxicity and patient quality of life is warranted.

## Conclusion

In conclusion, our study revealed the hematological differences among cisplatin-, nedaplatin-, lobaplatin- and nimotuzumab-based concurrent chemoradiotherapy for locally advanced head and neck cancers. Choice of concurrent regimens should take into considerations of their potential toxicities, patient tolerability, economic concerns and should pay specific attention to treat-related side effect that fits each regimen delivered.

## Data Availability Statement

The original contributions presented in the study are included in the article/**Supplementary Material**. Further inquiries can be directed to the corresponding authors.

## Ethics Statement

The clinical research projects were approved by Zhongnan Hospital of Wuhan University (Clinical Research Ethics Number: 2019130). Written informed consent to participate in this study was provided by the participants’ legal guardian/next of kin.

## Author Contributions

Conceptualization, QW, CZ, YFZ, and YHZ. Methodology, QW, CZ, YFZ, and YHZ. Software and data analysis, QW and CZ. Validation, QW, CZ, YFZ, and YHZ. Writing, review, and editing, QW, CZ, SZ, YFZ, and YHZ. Supervision, YFZ and YHZ. All authors have read and agreed to the published version of the manuscript.

## Funding

This study was supported by a grant from the Leading Discipline Construction Project of Oncology of Zhongnan Hospital of Wuhan University, a grant from the Scientific Research Project of Hubei Provincial Health and Family Planning Commission (grant no. WJ2019H064) and a grant from the Science, Technology and Innovation Seed Fund of Zhongnan Hospital of Wuhan University (Grant no. znpy2018123).

## Conflict of Interest

The authors declare that the research was conducted in the absence of any commercial or financial relationships that could be construed as a potential conflict of interest.

## Publisher’s Note

All claims expressed in this article are solely those of the authors and do not necessarily represent those of their affiliated organizations, or those of the publisher, the editors and the reviewers. Any product that may be evaluated in this article, or claim that may be made by its manufacturer, is not guaranteed or endorsed by the publisher.

## Abbreviations

LAHNC, locally advanced head and neck cancers; CTCAE, the Common Terminology Criteria for Adverse Events; CCRT, concurrent chemoradiotherapy; IMRT, intensity-modulated radiotherapy; DC, docetaxel plus cisplatin or nedaplatin; DCF, docetaxel plus cisplatin or nedaplatin, and 5-FU; GP, gemcitabine plus cisplatin of nedaplatin; WBC, white blood cell; HGB, hemoglobin; PLT, platelet; NEUT, neutrophils; SD, standard deviation; IQR, interquartile range; G-CSF, granulocyte colony stimulating factor; GM-CSF, granulocyte-macrophage colony stimulating factor.
