# Supplementary material for: Hematological Toxicities of Concurrent Chemoradiotherapies in Head and Neck Cancers: Comparison Among Cisplatin, Nedaplatin, Lobaplatin, and Nimotuzumab
Source: Front Oncol. 2021 Oct 21;11:762366. doi: 10.3389/fonc.2021.762366 (PMC8566976; doi:10.3389/fonc.2021.762366)
Supplement: Supplementary file 1 [file DataSheet_1.zip › Revised Supplementary materials.docx]

Table S1 The median cycles of treatment delivered with each drug and the proportion of patients on each treatment actually received chemotherapy cycles

|  |  | **Nimotuzumab** | **Cisplatin** | | **Nedaplatin** | | **Lobaplatin** |
| --- | --- | --- | --- | --- | --- | --- | --- |
|  |  | weekly (n=34) | weekly (n=21) | tri-weekly (n=31) | weekly (n=43) | tri-weekly (n=19) | tri-weekly (n=33) |
| **Median cycle (range)** | | 6 (3-8) | 4 (1-5) | 2 (1-2) | 4 (1-6) | 2 (1-3) | 2 (1-3) |
| **Cycles completed (ratio)** | 1 cycle | 0 | 2 (9.5%) | 9 (29.0%) | 2 (4.7%) | 4 (21.0%) | 12 (36.4%) |
|  | 2 cycles | 0 | 2 (9.5%) | 22 (70.1%) | 4 (9.3%) | 14 (73.7%) | 18 (54.5%) |
|  | 3 cycles | 3 (8.8%) | 3 (14.3%) | 0 | 4 (9.3%) | 1 (5.3%) | 3 (9.1%) |
|  | 4 cycles | 5 (14.7%) | 4 (19.0%) | NA | 13 (30.2%) | NA | NA |
|  | 5 cycles | 8 (23.5%) | 10 (47.6%) | NA | 14 (32.6%) | NA | NA |
|  | 6 cycles | 10 (29.4%) | 0 | NA | 6 (14.0%) | NA | NA |
|  | 7 cycles | 7 (20.6%) | 0 | NA | 0 | NA | NA |
|  | 8 cycles | 1 (2.9%) | 0 | NA | 0 | NA | NA |

NA: not applicable.

Table S2 Myelosuppression in patients treated with weekly or tri-weekly concurrent chemotherapy

|  | **Weekly concurrent chemotherapy arm (n=86)** | | | | | **Tri-weekly concurrent chemotherapy arm (n=61)** | | | | | | **P value** |
| --- | --- | --- | --- | --- | --- | --- | --- | --- | --- | --- | --- | --- |
|  | Grade 0 | Grade 1 | Grade 2 | Grade 3 | Grade 4 | | Grade 0 | Grade 1 | Grade 2 | Grade 3 | Grade 4 |  |
| **White blood cell** |  |  |  |  |  | |  |  |  |  |  |  |
| Cisplatin | 3 (14.3%) | 5 (23.8%) | 9 (42.9%) | 4 (19%) | 0 | | 2 (6.5%) | 4 (12.9%) | 16 (51.6%) | 9 (29%) | 0 | 0.489 |
| Nedaplatin | 1 (2.3%) | 10 (23.3%) | 15 (34.9%) | 12 (27.9%) | 5 (11.6%) | | 2 (10.5%) | 1 (5.3%) | 12 (63.2%) | 4 (21.1%) | 0 | 0.056 |
| **Neutrophil** |  |  |  |  |  | |  |  |  |  |  |  |
| Cisplatin | 10 (47.6%) | 6 (28.6%) | 2 (9.5%) | 3 (14.3%) | 0 | | 6 (19.4%) | 11 (35.5%) | 9 (29%) | 4 (12.9%) | 1 (3.2%) | 0.158 |
| Nedaplatin | 13 (30.2%) | 12 (27.9%) | 6 (14.0%) | 7 (16.3%) | 5 (11.6%) | | 5 (26.3%) | 5 (26.3%) | 7 (36.8%) | 2 (10.5%) | 0 | 0.261 |
| **Hemoglobin** |  |  |  |  |  | |  |  |  |  |  |  |
| Cisplatin | 8 (38.1%) | 7 (33.3%) | 4 (19%) | 2 (9.5%) | 0 | | 12 (38.7%) | 11 (35.5%) | 5 (16.1%) | 3 (9.7%) | 0 | 0.999 |
| Nedaplatin | 21 (48.8%) | 9 (20.9%) | 8 (18.6%) | 5 (11.6%) | 0 | | 11 (57.9%) | 7 (36.8%) | 1 (5.3%) | 0 | 0 | 0.175 |
| **Platelet** |  |  |  |  |  | |  |  |  |  |  |  |
| Cisplatin | 15 (71.4%) | 5 (23.8%) | 0 | 1 (4.8%) | 0 | | 21 (67.7%) | 6 (19.4%) | 3 (9.7%) | 1 (3.2%) | 0 | 0.554 |
| Nedaplatin | 19 (42.2%) | 4 (9.3%) | 8 (18.6%) | 12 (27.9%) | 0 | | 14 (73.7%) | 2 (10.5%) | 3 (15.8%) | 0 | 0 | 0.031 |
